# Preventing Patient Falls Overnight Using Video Monitoring: A Clinical Evaluation

**DOI:** 10.3390/ijerph192113735

**Published:** 2022-10-22

**Authors:** Rebecca Woltsche, Leanne Mullan, Karen Wynter, Bodil Rasmussen

**Affiliations:** 1 Directorate of Nursing & Midwifery, Western Health, 176 Furlong Road, St. Albans, VIC 3021, Australia; 2 School of Nursing and Midwifery, Deakin University, 1 Gheringhap St., Geelong, VIC 3220, Australia; 3 School of Nursing, Midwifery and Paramedicine, Australian Catholic University, 1100 Nudgee Road, Banyo, QLD 4014, Australia; 4 Centre for Quality and Patient Safety Research in the Institute for Health Transformation—Western Health Partnership, Deakin University, 1 Gheringhap St., Geelong, VIC 3220, Australia; 5 Faculty of Health and Medical Sciences, University of Copenhagen, Blegdamsvej 3B, 2200 Copenhagen, Denmark; 6 Faculty of Health Sciences, University of Southern Denmark and Steno Diabetes Center, Campusvej 55, 5230 Odense, Denmark

**Keywords:** accidental falls, hospitals, prevention and control, video monitoring, portable, falls prevention, inpatient falls, injuries

## Abstract

Inpatient falls are devastating for patients and their families and an ongoing problem for healthcare providers worldwide. Inpatient falls overnight are particularly difficult to predict and prevent. The aim of this cohort study was to evaluate effectiveness of overnight portable video monitoring as an adjunct falls prevention strategy for high falls risk patients in inpatient clinical units. Over three months, three clinical inpatient wards were provided with baby monitor equipment to facilitate portable video monitoring. Portable video monitoring registers were completed nightly and nursing staff were invited to complete surveys (n = 31) to assess their experiences of using portable video monitoring. A total of 494 episodes of portable video monitoring were recorded over the three-month period, with clinical areas reporting a total of four inpatient falls from monitoring participants (0.8% of total portable video monitoring episodes). Overall, there was a statistically significant reduction in total inpatient falls overnight on the target wards. Surveyed nursing staff reported feeling better equipped to prevent falls and indicated they would like to continue using portable monitoring as a falls prevention strategy. This study provides evidence to support the use of portable video monitoring as an effective falls prevention strategy in the hospital environment.

## 1. Introduction

Inpatient falls continue to be a serious patient safety problem for healthcare providers [1]. Up to one million falls occur in hospitals in the United States of America (USA) annually [2,3] and near 250,000 falls occur in inpatients settings each year in England alone [4]. Data from the United Kingdom (UK) indicates an average of 6.63 falls per 1000 occupied bed days [5] and rates of inpatient falls have been reported to be up to 9.1 per 1000 patient-days in geriatric hospital wards [6]. Between 2015–2016, there were more than 34,000 separations in which a patient was treated for injuries arising from an inpatient fall in Australian hospitals, a rate of 3.2 per 1000 separations [7]. Furthermore, the Australian Council on Healthcare Standards estimates that approximately 1800 hospital acquired falls result in fracture or intracranial injury in Australian hospitals every year [8] whilst 30% to 50% of falls in the USA are reported to result in injury [1].

The ageing population is growing faster than all other age groups globally [9]. The World Health Organisation anticipate one in six people will be aged over 60 years by 2030 and the number of people aged 80 years or older is expected to triple between 2020 and 2050 [10]. One in four people living in North America and Europe will be aged 65 or older by 2050 [11]. Australia’s population is also ageing with 19% predicted to be 65 years and over by 2031 [12] and by 2042, the number of Australians aged 85 years and over it estimated to double [13]. Risk of serious injury resulting from a fall is highest amongst older people and within the USA, up to 30% of older people who fall overall suffer moderate to severe injuries including hip fractures, head trauma, bruising or skin tears [14]. In a study analysing 3705 reported inpatient falls cases, it was found that the average age of a patient who fell was 68.5 years and those aged 65–74, 75–83 and >84 years were 19.5%, 29.3% and 39.1%, respectively, more likely to fall at a higher severity level [15]. With the growth of the aging population and the inpatient population containing higher numbers of older people, the challenge of preventing inpatient hospital falls and minimising falls severity will remain an issue for healthcare providers in the foreseeable future.

Economic costs of inpatient falls are also of concern. A study by Morello et al. [16] demonstrated that patients who fall in an acute hospital in Australia have an eight-day mean increase in length of stay and double the hospitalisation costs compared with non-fallers. The cost and management of these falls must be absorbed by the healthcare provider. Patients who experience inpatient falls resulting in injuries such as fractures or intercranial injury can remain in hospital for an average of almost 19 days longer than those who do not sustain such injury, due to severity of injuries [17], equating to $38,991AUD in additional costs [12].

The emotional impact inpatient falls have on healthcare staff was reported by Rush et al. [18] who identified that nurses often feel guilty when a patient falls, triggering self-doubt about their ability to deliver safe care. Nurses have been reported to be emotionally impacted after an inpatient fall and feel responsible for falls yet find it difficult to prevent falls [19]. Research involving 27 certified nursing assistants and registered nurses working in hospitals in the USA reported pressure from middle to senior level administrators to meet the goal of “zero falls” resulting in nurses having a fear of falls and thus restricting patient mobility [20]. In a study investigating the association between falls and bed moves, 486 Australian emergency department admissions were tracked. Data from 105 staff surveys and two focus groups involving 21 staff identified time pressures, poor communication between wards, limited room availability near the nurses’ station and bed movement to contribute to falls risk [21].

The physical, economic, and emotional implications of inpatient falls, combined with the increasing risk of falls due to the ageing population globally, prompts the need to review and implement innovative falls prevention strategies. There are several falls prevention strategies utilised in hospital settings including falls risk identification, alarm systems, sensors, patient education, environmental modifications, and non-slip socks [22]. High quality evidence supporting the efficacy of such interventions, either on their own or in a bundle, is, however, limited [22]. A randomised control trial (RCT) involving 24 wards across six Australian hospitals (n = 46,245 admissions), evaluated the effect of implementing the 6-PACK programme in comparison to usual care [23]. The 6-PACK programme involved utilising a falls risk assessment tool and one or more of six interventions, including ensuring walking aids were within reach of the patient, patient supervision, toileting regimens, use of low beds, bed/chair alarms and signage. This trial found that the rates of falls and injuries associated with falls were similar in both the control and intervention wards [23].

Further, despite Medicare implementing a non-payment policy for injuries related to inpatient falls, in 2008 in the USA, no reduction in injurious falls have been observed, indicating that interventions and changes to hospital practices have not improved outcomes [24]. In a 2018 Cochrane review, summation of 95 trials (n = 138,164 participants) found limited or low-quality evidence to support exercise, physiotherapy, bed sensor alarms, general medication review and multifactorial falls interventions to reduce risk and rates of falls [25]. The effectiveness of alarms has also been reviewed and RCTs in the USA and UK both found alarms to have no significant effect on falls in hospital settings [26,27]. A literature review examining the effectiveness of non-slip socks on falls prevention found inconclusive evidence supporting non-slip sock use as a falls prevention strategy amongst hospitalised older adults [28].

In a four-year observational study in Italy, fall patterns over a 24 h period were examined [29]. Fall occurrence was highest at night (46%), compared to morning (30%) or afternoon (24%) [29]. Overnight monitoring of falls risk is challenging due to lower staffing and patient-nurse ratios, and the potential for patients to attempt to mobilise independently, with minimal lighting and inadequate footwear [29]. With current falls prevention strategies lacking efficacy and reduced staffing levels at peak fall times, innovative interventions are needed to address falls amongst inpatients.

Recently, several research studies have demonstrated that video monitoring patients at risk of falling is an effective falls prevention strategy [30,31,32,33]. Cournan and colleagues [30], monitored 15 video monitoring units installed in high-risk units in a 115-bed inpatient rehabilitation facility, over a 21-month period. After a year of video monitoring use, falls had statistically significantly reduced from 6.34 to 5.10 falls per 1000 patient-days. A pre and post centralised video monitoring study conducted in 2013 also saw a significant reduction in falls from 3.9 to 2.8 falls per 1000 patient-days post implementation of a centralised video monitoring system [32]. A further study added a dedicated tele-sitter to a central video monitoring unit 24 h a day to observe up to 12 patients who had been identified to be at high risk of falls, within a 350-bed urban hospital [33]. The tele-sitter replaced a patient companion or sitter. Comparing falls data at baseline and 9-months post tele-sitter intervention, there was a significant reduction in the number of falls from 85 to 53 falls per patient discharge (*p* < 0.0001), a 35% reduction [33].

These studies all involved centralised fixed video monitoring together with additional technical staff employed to monitor the footage 24 h a day. Whilst Cournan et al. [30] indicated cost savings through reduction of falls and recouping initial cost outlay within 12-months of use, centralised video monitoring requires substantial infrastructure and budget to establish and maintain. Sand-Jecklin and colleagues [32] identified centralised video monitoring to reduce the number of sitter shifts by 23.2% and Votruba et al. [33] indicated sitter and falls-costs reduction to almost offset costs of centralised video monitoring and 24/7 tele-sitting staffing costs.

Centralised video monitoring, does however, rely on monitoring technicians maintaining good communication with ward staff [31]. In a study evaluating the video monitoring processes at a large teaching hospital, video monitoring technicians located in a centralised monitoring area reported difficulties in communicating with ward staff. Additionally, technicians did not feel equipped to respond to some patients such as those with drug use issues [31]. To date there is limited research reporting the utilisation of portable video monitors (PVM) for the prevention of falls among high falls risk (HFR) patients in acute care settings; that is, video monitoring that does not require additional staff to continuously observe monitors, nor need a centralised video monitoring system. To address the communication barriers identified in having a centralised video monitoring unit and technicians, as well as initial and human resource costs involved with centralised monitoring, this study aimed to: (a) evaluate effectiveness of overnight PVM as a falls prevention strategy for HFR patients in inpatient clinical units and (b) investigate nurses’ perceptions of PVM.

## 2. Materials and Methods

### 2.1. Design

The project commenced in April 2021. The health service deemed it feasible to conduct the study in three clinical inpatient wards in two tertiary hospitals in Melbourne, Australia for a period of three months. Video monitoring, both portable and centralised, has never been utilised within the health service involved in this study. Prior to the implementation of PVM, in an effort to minimise falls, nurses would complete hourly rounding where possible and answer patient call bells as required. The total number of bed days (n = 12,323) in the previous three-month period in the three clinical inpatient wards allowed for testing for statistically significant differences. These wards included a 24 bed Geriatric Evaluation and Management unit (Ward A), a 23 bed Acute Aged Care Medical ward (Ward B) and a 30 bed Acute Aged Care unit (Ward C). Project wards were selected as they had experienced high inpatient falls rates due to their patient cohorts and the wards had limited capacity to manage multiple HFR patients close to the nurses’ station or in high visibility rooms.

Each project ward was allocated baby monitor sets, including three monitors and six cameras, PVM registers and resource folders. Resource folders contained equipment manuals, troubleshooting tips, paper consent forms for families/patients, PVM information sheets for families/patients if requested and contact numbers for the project team. Multiple education sessions were conducted over Zoom, training the majority of nursing staff on the PVM process and equipment use. The equipment used in this PVM study was Uniden Baby Monitors—BW 3102R. Each monitor screen had the capacity to display up to four patients. The PVM detected movement and alerted the nurse via sound and image on the screen. Staff were advised to ensure the volume on the camera at the bedside was switched to full and the volume on the video monitor was also switched on. Any movement or rustling of sheets created a noise and alerted the nurse. The study was conducted overnight, with wards being much quieter than during the daytime, enabling staff to hear any movement on the PVM.

### 2.2. Participants

This was a cohort study design, following strobe recommendations, with all inpatient participants identified during routine falls risk assessments completed each shift by bedside nurses. Patients assessed as HFR on admission to a ward/unit who demonstrated confused and/or impulsive behaviours and were not specialled (allocated a one-on-one sitter/nurse) or situated in high visibility rooms, were eligible to be included in project. Verbal consent was obtained from either the patient, or their next of kin if the patient was unable to provide consent due to impaired cognition. PVM information sheets and information about the study was made available for families/patients to ensure informed consent was obtained. Completed consent forms were placed in a Project Falls Folder on each ward and an Electronic Medical Record (EMR) entry was made in the patient’s file stating consent for PVM had been obtained. An EMR order was created requesting PVM to commence at 8 p.m. each night as a reminder task for the evening nurse to attend to.

### 2.3. Data Collection

Once consent was obtained and documented in the patient’s EMR, a camera was set up in the patient room. The PVM remained at the bedside, in place for the duration of the time the patient was video monitored, however it would be switched off at 7:30 a.m. and switched back on between 8–9 p.m., prior to night staff arriving on shift at 9 p.m. PVM was not operational during the day and early evening hours. Nurses were able to take the PVM with them throughout their shift, improving their observational capacity to monitor their HFR patients. At the end of each night shift, nurses completed the PVM register (Supplementary Material A), recording the number of times they attended patients overnight as a result of the PVM, the reasons they attended the patients as a result of being alerted via the PVM, and whether they took the monitor with them during their patient rounds. There were no formal guidelines for discontinuing patient PVM, this was left to the discretion and clinical judgement of nursing staff.

The data sources used to measure the effectiveness of PVM were:

(1) PVM Register- results were collected weekly and entered into REDCAP (a database system) (Supplementary Material B)
(2) Map 2.0 and Riskman Incident reporting system to collect details of reported falls (Supplementary Material C)
(3) REDCAP staff survey with five fixed response questions and three open-ended questions to gather opinions and feedback for improvement (Supplementary Material D)—all staff recorded as working on nightshift during the trial period on any of the three participating wards were invited to participate in the staff survey. The survey was emailed to all staff to be printed off and manually completed then posted into a sealed box on each of the wards. The survey responses were collected after three weeks, and results entered into REDCAP.

### 2.4. Ethical Considerations

This study was submitted to the Western Health Research Ethics and Governance, Office for Research whereby the study was deemed exempt from Human Research Ethics Committee (HREC)/Low Risk Ethics Panel (LREP) review. The study was identified to be consistent with the National Health and Medical Research Council’s (NHMRC) Ethical Consideration in Quality Assurance and Evaluation Activities (2014) guideline and therefore approval and governance authorisation for the study was obtained across three hospital sites in Melbourne, Victoria, Australia.

### 2.5. Data Analysis

Descriptive statistics were applied to summarise quantitative data from the survey PVM register, Map 2.0 and Riskman incident reporting system, including frequencies and percentages. Bed occupancy for the pilot wards was extracted from the health service’s EMR for the three-month period before and during the implementation of the pilot. “Bed days” refer to the number of occupied beds at midnight on each date during that period. For the pre- and post-implementation time periods, the number of falls is expressed as a proportion of the number of bed days in each ward. Two-sample *z*-tests were used to compare the proportions of falls post-implementation, compared to pre-implementation.

Qualitative data from responses to open-ended questions in the survey were managed using an Excel spreadsheet and a manual text analysis was conducted with (a) broad-brush line-by-line coding, (b) development of themes (positive aspects of PVM, negative aspects of PVM and suggestions for improvement) which were then (c) cross checked by two members of the research team, ensuring credibility of thematic analysis. The first researcher was a staff member from the hospital, experienced in the management of patients at high risk of falls, having experience and understanding of the concepts being discussed. Therefore, the importance of reflexivity was acknowledged with all researchers and authors maintaining a reflective attitude to balance pre-existing beliefs, experiences, and attitudes, that may have impacted on analysis of open-end question responses and therefore the emergent data. No fixed number of participants was required in qualitative data collection, with participant suitability and accessibility being more significant.

## 3. Results

The total number of patient falls overnight on each ward reduced with the implementation of PVM. Ward A experienced the largest reduction in falls from a total 25 falls overnight pre PVM implementation down to seven falls post PVM implementation (reduction of 72%). Ward B successfully reduced their fall rate overnight by 50% from 16 to eight, followed by Ward C reducing total falls overnight from 15 to 10 (33%) (Table 1). The unwitnessed fall rate was also reduced in each of the clinical areas. Table 1 illustrates the significant decrease (*p* = 0.003) in total falls overnight with the implementation of PVM. Total falls reduced from 4.54 to 2.26 falls per 1000 bed days. There was a reduction in the number of unwitnessed falls in each area, and an increase in number of witnessed falls in the post PVM implementation period. PVM was not utilised during day and afternoon shifts and fall rates during these shifts remained constant or increased pre and post the PVM intervention (Table 1).

Across the three wards, 494 episodes of PVM (an episode of PVM is defined as one night of monitoring) were recorded over three months. No patients were reported to decline PVM during the trial. All patients were at high risk of falling. No other patient demographic data was collected. Out of the 494 episodes, there were four falls recorded; these included one unwitnessed fall, one Roll Out of Bed (ROOB) and two witnessed falls (in the presence of nursing staff). This is illustrated in Table 2 and equates to 0.8% of the total 494 episodes. A total of 1654 nursing interventions over the three clinical areas was recorded by nurses due to PVM alerting staff (Table 2). Staff in ward A were less likely to take the PVM on nursing rounds (34.8% vs. 85.4% and 92.9%) and be alerted by PVM in comparison to staff in wards B and C (Table 2).

There was a high number of additional interventions provided by nursing staff once alerted via the PVM that the patients were restless (Table 3). Nursing staff appeared to use this opportunity to identify and attend to a range of care needs, shifting the focus from reactive nursing care to preventative nursing principles. Toileting was the most frequent intervention provided by nursing staff, making up over 50% of all interventions across the three clinical areas.

Out of 48 nursing staff invited to complete the nurse experience survey, 31 (65%) submitted completed surveys (Table 4). Ninety seven percent of staff indicated that they enjoyed using PVM and would like to continue using the technology overnight, with 87% claiming they believe PVM has prevented patient falls overnight.

Open ended staff survey feedback was predominantly positive, maintaining that PVM was a helpful and valuable tool that was easy to implement and use.

*‘(I am) able to safely monitor multiple impulsive patients at the same time’*

*‘(PVM) alerts me to when a patient is getting up’*

*‘I like being able to see patients not in high visibility rooms’*

*‘Great we can carry PVM with us and monitor patients even when we are busy, (it) reduces (the) risk of patient(s) falling’*

When asked what they did not like about PVM, most of the nurses’ commentary related to the actual monitors and cameras that were used; specifically monitor screens that were too small, difficulty identifying patients and/or room numbers on such a small screen, limited battery life of monitors and cameras and some connectivity issues.

*‘Battery runs out quickly’*

*‘Camera sometimes goes out of range’*

*‘Can be hard to set up in some rooms’*

*‘Screen too small’*

*‘Difficult to monitor and get to patient when busy with another patient’*

Suggestions for improvement from staff included additional education, a slightly larger portable monitor screen to improve visualisation of two or more patients, longer battery life for the camera and monitor, and increased motion sensor capability to trigger an alert when the patient starts moving and attract the attention of the nurse.

## 4. Discussion

This study aimed to evaluate the effectiveness of overnight PVM as an adjunct falls prevention strategy for HFR patients in inpatient clinical units. Results are positive, with evidence of an overall significant fall reduction overnight. These findings reflect those of studies examining the value of centralised video monitoring. In a study involving COVID-19 patients in isolation, three video cameras were installed in patient rooms and a centralised monitor was placed at the nurse’s station [34]. Fall data collected over a four-month period indicated fall rates decreased by 45.5%. Additionally, 68% of survey respondents (n = 37), including nurses and patient care technicians, reported that they prevented between one and three falls whilst utilising video monitoring [34]. This study, much like the current study did not utilise a full-time technician or tele-sitter to monitor the centralised system, yet still observed a reduction in falls. 

Video monitoring has been found to be less expensive than sitters [35]. In a study involving 71 hospitals in the USA in 2017 and 2018, centralised video monitoring of 15,021 patients for a total of 942,482 h occurred. The human resource cost savings in this study equated to a reduction of 92%, from 453 to 38 annualised full-time equivalents required to support video monitoring [36]. Despite projected cost savings of centralised video monitoring over time, initial infrastructure outlay, maintenance, and additional staffing requirements [30,32] may be a barrier to implementation. The cost efficiencies of PVM present an enabler to initiating video monitoring. The successful application of widely available PVM technology to assist in fall reduction, without the considerable resources required for fixed centralised video monitoring supports PVM utilisation above centralised video monitoring. With an average cost of $480AUD for two cameras and one monitor (Supplementary Material E), PVM is an affordable intervention for healthcare facilities. Given the cost efficiencies and success of this study, a larger scale roll-out of PVM has occurred across the study’s health service’s sites.

The aforementioned study by Quigley et al., [36] identified 59 falls to occur during the study period and patients who experienced a fall received 20.5 verbal interventions before a fall from the monitoring staff per patient day, whilst those who did not fall received 15.7 verbal interventions. This indicates the significant intervention required to support patients who are at risk of falling [36]. Interestingly, despite the potential additional burden such interventions could place on nursing staff, the current study identified improved nurse satisfaction with the implementation of PVM. A study by Bok et al. [37] illustrated the positive effects that the known prevention of inpatient falls can have on nursing staff, and the subsequent negative impact an inpatient fall can have on nurse confidence, anxiety and satisfaction in the workplace. Nurses reported that they felt ‘horrible’, ‘guilty’ and that they had ‘failed to protect the patient’ when detailing their feelings in response to a patient fall [37] (p. 49). When strategies failed to prevent a patient’s fall, nurses in Rush and colleagues’ study reported that they experienced considerable stress, describing falls as ‘scary’ and ‘upsetting’ [18]. The uncertainty surrounding potential injury, time consuming nature of post fall protocol, logistics of moving a patient post fall and potential liability have also been reported as stresses for nurses working in inpatient hospital units [18]. Nurses in the current study reflected that they genuinely appreciated being able to identify and respond to patients before they fell, indicating their alignment with the studies regarding their desire to reduce the potential stressors related to falls.

Reluctance of some staff to embrace new technology may be a factor for consideration with the initial uptake and ongoing implementation of PVM. Within this study, respondents from ward A were less likely to take PVM with them on their nursing rounds and therefore received fewer PVM alerts. Research into nurses’ reactions to novel technology in acute health care has identified barriers to uptake to relate to perceived threats to clinical skills and limited capability of technology to capture clinical workflow, whilst enablers relate to the technology being perceived to help management and support of nursing processes [38]. Involving nursing staff early in the implementation of new technology such as PVM may encourage adoption of the system [38]. Additionally, insufficient, or ineffective digital education has been reported to result in nurses digitally lagging, influencing adoption of health information technologies negatively [39]. Staff in the current study expressed they would have liked additional training sessions for PVM throughout the trial period. Capturing and educating all nursing staff on how to use PVM and troubleshoot issues is often challenging, due to varying staff shifts and workloads. Creating educational videos for staff to access anytime is an appropriate and potentially effective solution to this problem. Ensuring the content of training and education is suitable to the needs of staff has been recommended to support adoption of new health technologies [39].

It is noted that falls were not eliminated with the use of PVM, with 25 falls occurring overnight during the study period, with four of these being falls in patients who were being monitored by PVM. As there was no-one employed to continuously observe monitors, at times, nurses may have been attending to another patient and not been looking at the monitor screen. PVM is an additional strategy to extend nurses’ observations of HFR patient and supports staff to make informed decisions regarding which patients they attend to and in what order. Video monitoring technology for fall prevention is advancing rapidly, with the development of artificial intelligence (AI) related falls prevention strategies underway [40,41]. PVM fills the gap until AI, including AI predictive technology and computer vision technics become more widely available and financially accessible to healthcare providers. PVM can be purchased off the shelf, is widely accessible, very affordable and can be easily implemented without changes to infrastructure. Additionally, new monitors are being added to the market which may address limitations of current devices.

## 5. Strengths and Limitations

To the authors’ knowledge, this is the first study to examine the effectiveness of PVM on inpatient falls reduction. Additionally, the study garnered the opinions of staff on utilising PVM and whether they would support continued PVM use.

Limitations of using ‘off the shelf’ commercial baby monitors, not designed for use in hospitals as a falls prevention strategy, may be one study limitation, as highlighted by responses to open-ended questions regarding small screen size, and thus a barrier to wide-spread uptake of PVM in acute care settings. Whilst the monitors used in this trial were effective, a review of all available commercial monitors, to identify which are most suitable for inpatient PVM, is recommended for future clinical application. Implementation of a larger screen, whilst helpful to visibility, would reduce portability of the PVM. Connecting the PVM to a larger centralised screen is an option for further investigation, however, on nightshift there are reduced nursing levels, thus potentially less chance that a staff member would be available to watch the centralised screen.

The sample size and the use of PVM in just three inpatient units are limitations of the study and as such findings cannot be generalised for the population. The findings do, however, contribute to the limited data on PVM and provide healthcare providers with evidence to support a low-cost video monitoring option.

## 6. Conclusions

Study results indicate a reduction in patient falls overnight across all three clinical areas. Staff survey results overwhelmingly support the use of PVM overnight, with nurses stating they believe it has helped them prevent inpatient falls and all, but one member of staff indicated they would like to continue using the technology. PVM is commercially accessible to all healthcare providers from multiple retailers. It is inexpensive to purchase, cost effective to use and easy to implement without requiring additional infrastructure. PVM has clearly established itself as an evidence-based falls prevention strategy and creates another helpful tool healthcare providers can use to prevent inpatient falls.

The current study identified the benefit of PVM as a falls prevention strategy in clinical inpatient units. Utilisation of PVM resulted in a reduction in the number of patient falls in three distinct wards and an overall decrease in the number of falls per 1000 bed days in comparison to pre-PVM use. PVM provides healthcare settings with a low-cost adjunct falls prevention strategy for HFR patients. Developing guidelines and supporting staff education for PVM use in clinical areas, implementation of PVM as an endorsed falls prevention strategy in clinical inpatient units and endorsing use of PVM when nursing suspected COVID-positive or COVID-positive patients due to isolation requirements, is recommended in addition to standard strategies to assist in preventing falls in clinical settings.

## Figures and Tables

**Table 1:** Overall comparison of total inpatient falls data overnight and pre and post PVM implementation.

Fall Classification	Ward A		Ward B		Ward C		Total	
	Pre PVM Nov-March	Post PVM April-Aug	Pre PVM Nov-March	Post PVM April-Aug	Pre PVM Nov-March	Post PVM April-Aug	Pre PVM Nov-March	Post PVM April-Aug
Witnessed falls overnight	0	0	3	2	0	5	3	7
Unwitnessed falls overnight	14	4	8	2	12	5	34	11
ROOB from Hilo bed onto crash mat—unwitnessed overnight	11	3	5	4	3	0	19	7
Total number of falls overnight	25 (55%)	7 (26%)	16 (47%)	8 (29%)	15 (44%)	10 (24%)	56	25
Total number of ‘bed days’ (beds occupied)	4124	3346	3448	3214	4751	4513	12323	11073
Falls/1000 ‘bed days’							4.54	2.26
Prop	0.006062	0.002092	0.00464	0.002489	0.003157	0.002216	0.0045443	0.002258
	Significant decrease (*p* = 0.009)		Not significant (*p* = 0.1431)		Not significant (*p* = 0.3829)		Significant decrease (*p* = 0.003)	
Total falls during day/evening shifts	20 (45%)	20 (74%)	18 (53%)	18 (71%)	19 (56%)	31 (76%)	57	69
Total falls per unit	45 (100%)	27 (100%)	34 (100%)	26 (100%)	34 (100%)	41 (100%)	113	94

**Table 2:** PVM register results.

Ward	Number of PVM Episodes	Number of Falls by Patients on PVM	Number. Times Staff Alerted via PVM	PVM Taken on Nursing Rounds
A	141	2 unwitnessed (1 ROOB)	317	Yes 49 (34.8%) No 92 (65.2%)
B	199	1 witnessed	478	Yes 170 (85.4%) No 29 (14.6%)
C	154	1 witnessed	859	Yes 143 (92.9%) No 11 (7.1%)
Totals	494	4 (0.8%)	1654	Yes 362 No 132

**Table 3:** PVM staff survey results-reasons reported for nurse attendance/intervention due to PVM.

Ward	Toileting	Thirst/Hunger	Pain	Other *	Total
A (2H)	54.4%	10.6%	4.4%	30.6%	100%
B (3B)	56.9%	4%	4%	35.1%	100%
C (2A)	56%	24.5%	8.4%	11.1%	100%

* Interventions reported as other were not always specified by the nurse. Some reasons reported related to patient restlessness and repositioning.

**Table 4:** Staff satisfaction survey results (n = 31).

Staff Survey Question	n (%) Responded ‘Yes’			
	Ward A	Ward B	Ward C	Total
Do you like using PVM overnight?	92% (13)	100% (10)	100% (7)	97% (30)
Have you been alerted to a patient as a result of the PVM?	57% (8)	100% (10)	100% (7)	80% (25)
Do you believe PVM have prevented patient falls overnight?	71% (10)	100% (10)	100% (7)	87% (27)

## Data Availability

The authors confirm that the data supporting the findings of this study are available within the article [and/or] its Supplementary Materials. Participants of this study did not agree for their data to be shared publicly, so additional supporting data is not available.

## Supplementary Materials

The following supporting information can be downloaded at: https://www.mdpi.com/article/10.3390/ijerph192113735/s1, Supplementary Material A: Video Monitoring Register; Supplementary Material B: Portable Video Monitoring Staff Survey REDCAP Database Register; Supplementary Material C: Riskman Incident Reporting System Database; Supplementary Material D: Portable Video Monitoring Overnight Staff Satisfaction Survey; Supplementary Material E: Uniden Baby Monitor Costing.

## Abbreviations

AI	Artificial Intelligence
AUD	Australian Dollars
EMR	Electronic Medical Record
HFR	High Falls Risk
HREC	Human Research Ethics Committee
LREP	Low Risk Ethics Panel
NHMRC	National Health and Medical Research Council
PVM	Portable Video Monitoring
RCT	Randomised Control Trial
ROOB	Roll Out of Bed
UK	United Kingdom
USA	United States of America
