# Supplementary material for: Preventing Patient Falls Overnight Using Video Monitoring: A Clinical Evaluation
Source: Int J Environ Res Public Health. 2022 Oct 22;19(21):13735. doi: 10.3390/ijerph192113735 (PMC9657748; doi:10.3390/ijerph192113735)
Supplement: Supplementary file 1 [file ijerph-19-13735-s001.zip › Supplementary Material A - Video Monitoring Register.pdf]

Supplementary Material A: Video Monitoring Register

Ward ____________________ Date ___________________

Patient Label	Bed No	Reason for Monitoring (Tick All That Apply)	Record of Times Nurse Attended Patient after Being Alerted by Monitor	Did You Take PVM on Patient Rounds
		€ Assessed as HFR using FRAT<br>€ Confused/not orientated<br>€ Impulsive<br>€ Other:…………………………	No: of times attended patient O/N: ________<br>Reason:<br>€ Toileting<br>€ Pain<br>€ Hunger/Thirst<br>€ Other :_________________	Yes/No
		€ Assessed as HFR using FRAT<br>€ Confused/not orientated<br>€ Impulsive<br>€ Other:…………………………	No: of times attended patient O/N: ________<br>Reason:<br>€ Toileting<br>€ Pain<br>€ Hunger/Thirst<br>€ Other :_________________	Yes/No
		€ Assessed as HFR using FRAT<br>€ Confused/not orientated<br>€ Impulsive<br>€ Other:…………………………	No: of times attended patient O/N: ________<br>Reason:<br>€ Toileting<br>€ Pain<br>€ Hunger/Thirst<br>€ Other :_________________	Yes/No
		€ Assessed as HFR using FRAT<br>€ Confused/not orientated<br>€ Impulsive<br>€ Other:…………………………	No: of times attended patient O/N: ________<br>Reason:<br>€ Toileting<br>€ Pain<br>€ Hunger/Thirst<br>€ Other :_________________	Yes/No
		€ Assessed as HFR using FRAT<br>€ Confused/not orientated<br>€ Impulsive<br>€ Other:…………………………	No: of times attended patient O/N: ________<br>Reason:<br>€ Toileting<br>€ Pain<br>€ Hunger/Thirst<br>€ Other :_________________	Yes/No
