# Supplementary material for: Preventing Patient Falls Overnight Using Video Monitoring: A Clinical Evaluation
Source: Int J Environ Res Public Health. 2022 Oct 22;19(21):13735. doi: 10.3390/ijerph192113735 (PMC9657748; doi:10.3390/ijerph192113735)
Supplement: Supplementary file 1 [file ijerph-19-13735-s001.zip › Supplementary Material B - Portable Video Monitoring Staff Survey REDCAP Database Register.pdf]

Supplementary Material B: Portable Video Monitoring Staff Survey REDCAP Database Register

1) Date ______________________________

2) Ward
☐ 1. 2H Sunshine Hosp
☐ 2. 3B Footscray Hosp
☐ 3. 2A Sunshine Hosp

3) UR ______________________________

4) Falls Risk Category
☐ High Falls Risk
☐ Moderate Falls Risk
☐ Low Falls Risk

5) PVM Criteria
☐ HFR
☐ Confused
☐ Impulsive
☐ NESB

6) Reason nurse attended patient due to PVM
☐ Toileting
☐ Pain
☐ Hunger / Thirst
☐ Other

7) If selected Other, please document reason: ______________________

8) Number of times nurse responded to patient after alerted by PVM __________

9) Was PVM taken by nursing staff on patient rounds
☐ Yes
☐ No

10) Did patient fall overnight
☐ Yes
☐ No

11) If patient fall, Riskman number ______________________

12) Additional notes ______________________________
