# Supplementary material for: Preventing Patient Falls Overnight Using Video Monitoring: A Clinical Evaluation
Source: Int J Environ Res Public Health. 2022 Oct 22;19(21):13735. doi: 10.3390/ijerph192113735 (PMC9657748; doi:10.3390/ijerph192113735)
Supplement: Supplementary file 1 [file ijerph-19-13735-s001.zip › Supplementary Material C - Riskman Incident Reporting System Database.pdf]

Supplementary Material C: Riskman Incident Reporting System Database

1) Riskman Number____

2) Ward
☐ 1. 2H Sunshine Hosp
☐ 2. 3B Footscray Hosp
☐ 3. 2A Sunshine Hosp

3) UR ID ____

4) Date ____

5) Time of fall ____

6) Type of fall
☐ Roll out of Lolo bed with crash mat insitu
☐ Witnessed fall no headstrike
☐ Witnessed fall – with headstrike
☐ Unwitnessed fall

7) PVM Insitu
☐ Yes
☐ No

8) Patient Falls Risk Category
☐ High
☐ Moderate
☐ Low
