# Supplementary material for: Preventing Patient Falls Overnight Using Video Monitoring: A Clinical Evaluation
Source: Int J Environ Res Public Health. 2022 Oct 22;19(21):13735. doi: 10.3390/ijerph192113735 (PMC9657748; doi:10.3390/ijerph192113735)
Supplement: Supplementary file 1 [file ijerph-19-13735-s001.zip › Supplementary Material D - Portable Video Monitoring Overnight Staff Satisfaction Survey.pdf]

Supplementary Material D: Portable Video Monitoring Overnight Staff Satisfaction Survey

Dear Nurse

Thank you for supporting the QA Project Preventing Patient Falls Overnight using Live and Portable Video Monitoring QA project Number: QA2021.25. We have collected data for 500 episodes of PVM across the three project areas. We now need YOUR opinions on using PVM overnight. Was it a help or a hindrance? Please complete the survey below. Your responses are important to the evaluation and potential ongoing use of PVM at Western Health.

1) Ward
   ☐ 1. 2H Sunshine Hosp
   ☐ 2. 3B Footscray Hosp
   ☐ 3. 2A Sunshine Hosp

2) Permanent N/S staff
   ☐ Yes – always work nightshift
   ☐ No – regularly work both days and night

3) Do you like using PVM overnight
   ☐ Yes
   ☐ No

4) Have you been alerted to a patient as a result of the PVM
   ☐ Yes
   ☐ No

5) Do you believe PVM have prevented patient falls overnight
   ☐ Yes
   ☐ No

6) What do you like about using PVM overnight ______________________________

7) What do not you like about using PVM overnight ____________________________

8) Do you have any suggestions to improve PVM overnight ______________________
