# Supplementary material for: Preventing Patient Falls Overnight Using Video Monitoring: A Clinical Evaluation
Source: Int J Environ Res Public Health. 2022 Oct 22;19(21):13735. doi: 10.3390/ijerph192113735 (PMC9657748; doi:10.3390/ijerph192113735)
Supplement: Supplementary file 1 [file ijerph-19-13735-s001.zip › Supplementary Material E - Uniden Baby Monitor Costing.pdf]

Supplementary Material E: Uniden Baby Monitor Costing

Q-36110 - MH Baby Monitor

Image SKU	Description	QTY	Sales Price Excl. GST	Sales Price Incl. GST	Subtotal Sales Price Incl. GST
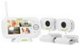 439248	Uniden BW3102 4.3' Digital Wireless Baby Video Monitor with Two Cameras<br>colour<br>**Manufacturer:** UNIDEN<br>**Model:** BW3102	2	$ 213.48	$ 234.83	$ 469.66
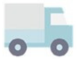 86499	Customer Freight Charge<br><br>**Manufacturer:** JB HI-FI<br>**Model:** 86499	1	$ 13.64	$ 15.00	$ 15.00

Total Sales Price Excl. GST $440.60

Total Sales Price Incl. GST $ 484.66
